# Probing the properties of PTEN specific botulinum toxin type E mutants

**DOI:** 10.1007/s00702-025-02879-2

**Published:** 2025-01-23

**Authors:** Giorgia Schiavone, Sandy Richter, Tina Henke, Ineke Koch, Linda Thies, Fiete Klöpper, Aram Megighian, Marco Pirazzini, Thomas Binz

**Affiliations:** 1 Department of Biomedical Sciences, University of Padova, Padova, 35131 Italy; 2 Institut für Zellbiochemie, OE 4310, Medizinische Hochschule Hannover, 30623 Hannover, Germany; 3 Padova Neuroscience Center, University of Padova, Padova, 35131 Italy

**Keywords:** SNAP-25, PTEN, Zinc protease, Botulinum neurotoxin, Neurotoxin engineering, Substrate specificity

## Abstract

Botulinum neurotoxins (BoNT) are established biopharmaceuticals for neuromuscular and secretory conditions based on their ability to block neurotransmitter release from neurons by proteolyzing specific soluble N-ethylmaleimide-sensitive factor attachment protein receptor (SNARE) proteins. Recently, a mutant catalytic domain of serotype E (LC/E) exhibiting 16 mutations was reported to cleave the phosphatase and tensin homolog (PTEN). This molecule represents an attractive new target in neurons as several reports support PTEN knockdown as a strategy to stimulate axonal regeneration after injury. Though this LC/E mutant was shown to cleave PTEN in primary neurons through lentivirus-based expression, its expression and functionality as mutated full-length BoNT/E have not been studied. Hence, we assembled the 16 mutations stepwise in a bacterial expression plasmid for LC/E and purified several multiple mutants of LC/E. Biochemical characterization showed that the 16-fold mutant did not exhibit a detectable activity toward SNAP-25 up to 10 µM final concentration while it displayed an EC_50_ of approximately 200 nM for PTEN, exceeding 1000-fold that for LC/E-wt on the native substrate SNAP-25. Unexpectedly, expression of the full length 16-fold mutated BoNT/E did not provide soluble protein, possibly due to an interference of the interaction between LC and the translocation domain. Reversion of individual mutations revealed the E159L and S162Q substitutions, critical for redirecting LC/E activity toward PTEN, as main culprits for the solubility issue. To overcome this problem, we applied a methodology proved successful years ago, harnessing a proteolytically inactive variant of BoNT type D (BoNT/Di) as neurospecific delivery system for cargo proteins. The fusion protein LCE-16x-BoNT/Di could be produced in sufficient yields. Activity tests using rat cerebellar granule neurons showed BoNT/E-like activity for LC/E-wt-BoNT/Di, but no PTEN-directed activity for LC/E-16x-BoNT/Di.

## Introduction

Botulinum neurotoxins (BoNTs) serve for more than 30 years as effective pharmaceuticals for treating neurological conditions caused by hyperactivity of cholinergic nerve terminals such as of blepharospasm, hemifacial spasm, and strabismus. Their range of applications now includes, besides a large variety of neuromuscular and autonomic disorders, aesthetic medicine applications and other non-neuronal uses (Jankovic 2017; Pirazzini et al. 2022; Pirazzini et al., 2017b). Based on their immunological properties, BoNTs have been classified as different toxin types referred to as serotypes that are indicated with an alphabetical letter (BoNT/A-/G, BoNT/H and BoNT/X) (Dong et al. 2019; Peck et al. 2017). Of the nine serotypes, only serotype A (BoNT/A), and for some indications BoNT/B, are approved for treatment and marketed as several commercial preparations (Chen 2012; Choudhury et al. 2021; Frevert 2015; Chen et al. 2012), while BoNT/E is presently in clinical trials (Lebowitz and Berson 2022; Pons et al. 2019; Yoelin and Hooper 2024).

BoNTs are synthesized as ~ 150 kDa single-chain proteins, which are subsequently cleaved into an enzymatic light chain (LC) of ~ 50 kDa and a heavy chain (HC) of ~ 100 kDa held together via a single disulfide bond and non-covalent interactions in particular between LC and the so-called belt region (Dong et al. 2019; Rossetto et al. 2014). This region represents the N-terminal part of HC and wraps around the LC. BoNTs exhibit an exquisite specificity for neuronal cells due to the HC mediated binding to polysialogangliosides and a synaptic vesicle protein that leads to endocytosis. Upon acidification of the endocytic vesicle the N-terminal half of HC (H_N_) forms a channel to deliver the LC to the cytosol involving the belt region in the translocation mechanism (Montal 2010; Pirazzini et al. 2016). Thereafter, the interchain disulfide bond is reduced by the thioredoxin-thioredoxin reductase couple, which releases the metalloprotease activity of the LCs against specific members of the neuronal SNARE (soluble N-ethylmaleimide-sensitive factor attachment protein receptors) family (Pirazzini et al. 2017a). These proteins constitute the core of the synaptic vesicle fusion machinery. E.g. BoNT/A and E hydrolyze SNAP-25 (synaptosomal-associated protein of 25 kDa), whereas BoNT/B cleaves synaptobrevin/VAMP (vesicle associated membrane protein) isoform 1, 2, and 3 (Pirazzini et al., 2017b). Disabling of one such SNARE blocks the fusion of neurotransmitter-containing synaptic vesicles, resulting in flaccid paralysis and autonomic dysfunction, the hallmark symptoms of botulism (Johnson and Montecucco 2008). Despite severe cases can result in respiratory failure and potentially death (Dong et al. 2019; Pirazzini et al. 2022), patient can survive with mechanical respiratory support and fully recover over time. In fact, BoNTs are not cytotoxic and their neuroparalytic effects are fully reversed once the LC is degraded by intracellular systems and cleaved SNARE proteins are replaced with newly synthesized ones. This reversibility of action, combined with limited diffusion from the injection site and highly selective neurotropism, underpins the exceptionally safe and effective use of BoNTs in both therapeutic and aesthetic medicine.

Current research addresses the expansion of the use of BoNTs to further medical conditions, including diseases associated with hypersecretion from non-neuronal cells, like the widespread chronic obstructive pulmonary disease, asthma or diabetes. In this scenario, use of BoNTs is impeded due to the resistance of respective SNAREs to BoNT LCs and the lack of appropriate receptor molecules on target cells. However, delivery of BoNT LCs to cells not targeted naturally by the toxins can be enabled by technologies like the targeted secretion inhibitor (TSI) platform or SNARE tagging technology (Darios et al. 2010; Fonfria et al. 2018). Both techniques combine engineered proteins consisting of the LC and H_N_ translocation domain, called LH_N_, and a binding domain that allows binding to a cell surface receptor on the desired target cell and subsequent entry into the endosomal compartment. The issue of the involvement of refractory SNAREs in secretion of non-neuronal cells has also been successfully processed. Screening methods combined with rational structure-driven design led to a BoNT/A quadruple mutant readily cleaving human SNAP-23 (hSNAP-23), an isoform of SNAP-25 being refractory to wild-type BoNT/A, in neuronal cell based assays (Sikorra et al. 2020).

Recently, Blum et al. developed an efficient method of LC mutagenesis based on a M13 phage-assisted evolution system, which allows applying both positive and negative selection constrains to redirect the protease specificity (Blum et al. 2021). By this method, the protease domain of BoNT/X was evolved into variants that preferentially cleave the VAMP family members VAMP4 or Ykt6. In addition, LC/F was evolved into a protease cleaving the non-native substrate VAMP7 (Blum et al. 2021). Furthermore, this approach created a LC/E derivative specifically cleaving phosphatase and tensin homolog (PTEN), but no longer its authentic substrate SNAP-25 (Blum et al. 2021).

PTEN is a ubiquitously expressed tumor suppressor gene with multifaceted roles regulating various cellular processes, including cell survival and growth through its modulation of signalling pathways, particularly the PI3K/AKT axis. In the nervous system, PTEN is highly expressed in neurons and performs crucial functions in neurodevelopment, neuron survival and regeneration (Duraikannu et al. 2019; Park et al. 2010). During neurodevelopmental neurite outgrowth, PTEN is inactivated through phosphorylation and sequestered away from the growth cone, leading to increased PIP3 levels on the axonal tip facilitating its elongation (Chadborn et al. 2006). Upon reaching the target, PTEN is recruited back on the growth cone and reactivated, promoting growth cone collapse and enabling the formation of synaptic connections (Chadborn et al. 2006; Henle et al. 2013). Given its established role as negative modulator of axonal growth during development, several lines of evidence ascribed PTEN as a major neuron intrinsic blocker of axonal regeneration. Consistently, PTEN knockdown or deletion was found sufficient to both enhance regeneration of damaged peripheral nerves and even to revive axonal regrowth of the adult central nerves, normally refractory to regeneration after injury (Christie et al. 2010; Liu et al. 2010; Meyer Zu Reckendorf et al., 2022; Park et al. 2008). On the other hand, non-targeted PTEN ablation can enhance cell proliferation in proliferating cells, increasing the risks of tumorigenesis (Suzuki et al. 2003; Zagni et al. 2017). Leveraging the exquisite neurotropism of BoNTs, we hypothesized that engineering a BoNT mutant carrying a PTEN-specific protease to biochemically knock down PTEN activity specifically into neurons could represent an effective strategy to foster axonal regeneration.

Here, we generated the PTEN-specific LC/E by stepwise assembling the underlying 16 mutations identified by Blum et al. (2021) and studied their individual contribution to the catalytic activity on PTEN and SNAP-25 by in vitro cleavage assays. Unexpectedly, expression and purification of the corresponding 16-fold mutated full-length BoNT/E failed, likely due to stability and solubility issues. We thus produced and analyzed a variety of intermediate full-length BoNT/E mutants finding that the mutations most critical for PTEN cleavage most strongly impair the structural stability of the 16-fold mutated full-length BoNT/E. To overcome the solubility issue and to generate a PTEN-specific BoNT derivative that can be applied exogenously to nerve cell preparations, we constructed fusion proteins in which the 16-fold mutated LC/E (or wild-type LC/E as a control) was appended as passenger to the N-terminus of a catalytically inactive full-length BoNT/D (BoNT/Di). The LC/E-wt-BoNT/Di and LC/E-16x-BoNT/Di fusion proteins could be purified from *E. coli* lysates and were thus tested in primary neuronal cultures. As assessed by monitoring SNAP-25 and PTEN cleavage, this strategy proved effective in delivering LC/E-wt, but not LC/E-16x, into the neuronal cytosol. It remains unclear whether LC/E-16x-BoNT/Di is unable to physically translocate its catalytic moiety into the neuronal cytosol or whether LC/E-16x is delivered in sufficient amounts to allow PTEN cleavage.

## Materials and methods

### Plasmid constructions

The cDNA for full-length human PTEN (403 amino acids; accession: NM_000314) was inserted into the in vitro transcription vector pSP73 (Promega, Mannheim, Germany) using the *Bam*HI and *Hin*dIII restriction sites yielding pSP73-hPTEN. The plasmid used for synthesis of mouse SNAP-25A by in vitro transcription/translation (pSNAP-25) has been described earlier (Binz et al. 1994). The BoNT/E LC (subtype 1, strain Beluga, aa 1-422) encoding DNA-fragment was ligated into the *Bam*HI and *Hin*dIII restriction sites of pQE9 (Qiagen, Hilden, Germany) resulting in pFB1. Mutants of pFB1 were generated by PCR applying the GeneTailor^™^ site-directed mutagenesis system (Thermo Fisher Scientific, Darmstadt, Germany) and suitable primers. All mutations were verified by sequencing (Eurofins Genomics, Ebersberg, Germany). The expression plasmid pH6BoNTEs-thro for production full-length BoNT/E in *E. coli* (Rummel et al. 2009) served as parental vector for generating mutated BoNT/E. Its LC encoding segment was replaced with those of the pFB1 mutants using the vector-based *Bam*HI and a *Swa*I site present near the 3’ end of the LC specific segment. pH6-LCE-wt-BoNTDs-inact was generated by inserting a *Xho*I/*Kpn*I cleaved LC/E encoding DNA fragment exhibiting a *Sal*I-site upstream of the *Kpn*I site generated by PCR into corresponding sites of pH6tBoNTDs-inact. The *Xho*I and *Sal*I sites of pH6-LCE-wt-BoNTDs-inact served then as cloning sites for the 16-fold mutated LC/E encoding DNA fragment to yield pH6-LCE-16x-BoNTDs-inact. pH6tBoNTDs-inact was derived from pASK-IBA3-BoNTD-E230A (Bade et al. 2004) by subcloning into pQE3 and introduction of the mutations R372A and Y375F by PCR.

### Production and purification of recombinant proteins

LC/E and its mutants, full-length BoNT/E, and LC/E-BoNT/D fusion proteins were produced using the *E. coli* strain M15[pREP4] (Qiagen, Hilden, Germany) during 15 h of induction at 21 °C in the presence of 0.25 mM IPTG. LC/E and its mutants were purified on Ni^2+^-nitrilotriacetic acid-agarose (NTA) beads, dialyzed against 10 mM HEPES/KOH, pH 7.2, supplemented with 150 mM potassium glutamate (toxin assay buffer), shock-frozen in liquid nitrogen, and stored at -70 °C until use. Full-length BoNT/E and LC/E-BoNT/D fusion proteins were subsequent to elution from NTA beads using 100 mM imidazole in addition purified on Strep-Tactin sepharose beads (IBA Lifesciences, Göttingen, Germany) and eluted with 10 mM desthiobiotin in 100 mM Tris, pH 8.0. Full-length BoNT/E and LC/E-BoNT/D fusion proteins were manufactured under biosafety level 2 containment (Bezirksregierung Hannover, project number A/Z 40654/3/57). Protein concentrations were determined following thawing of frozen aliquots, SDS-PAGE analysis and Coomassie blue staining by means of the LAS-3000 imaging system (Fuji Photo Film, Co., Ltd.) and the Multi Gauge software (Fuji Photo Film). Various known concentrations of bovine serum albumin were run as standard. PTEN and SNAP-25A were generated by in vitro transcription/translation via the above described plasmids, the SP6 coupled TNT reticulocyte lysate system (Promega, Walldorf, Germany), and L-[^35^S]methionine (370 kBq/µL, > 37 TBq/mmol, Hartmann Analytic, Braunschweig, Germany) according to the manufacturer´s instructions.

### Endopeptidase assays

Cleavage assays contained 2 µL of radiolabeled PTEN or SNAP-25A and 18 µl of LC/E or mutated LC/E at various final concentrations as specified in legends to figures and tables in toxin assay buffer. Protease reactions were run for 60 min at 37 °C and stopped by the addition of 20 µL of double-concentrated sample buffer [120 mM Tris-HCl, pH 6.75, 10% (v/v) β-mercaptoethanol, 4% (w/v) SDS, 20% (w/v) glycerol, 0.014% (w/v) bromphenol blue]. Samples were incubated at 99 °C for 2 min and then subjected to SDS-PAGE using 12.5% gels. Subsequently, gels were dried and radiolabeled proteins were visualized employing a FLA-9000 phosphorimager (Fuji Photo Film, Co., Ltd., Tokyo, Japan). Quantification of radiolabeled proteins and their cleavage products was done with Multi Gauge software (Fuji Photo Film).

Cleavage assays were performed also on brain lysates. Briefly, a mouse was intracardially perfused with 50 milliliters of PBS, the brain cortices were collected, cut in small pieces and mechanically homogenized in 320 mM sucrose, 4 mM HEPES (pH 7.3). After centrifugation, the supernatant was eliminated and the resulting brain membranes were solubilized in 1.5 milliliters of radio-immunoprecipitation assay (RIPA) buffer (10 mM Tris-HCl, pH 8.0, 1 mM EDTA, 0.5 mM EGTA, 1% Triton X-100, 0.1% Sodium Deoxycholate, 0.1% SDS, 140 mM NaCl). LC/E-16x or LC/E-BoNT/D fusion proteins were added to 20 µL of brain lysate and incubated at 37 °C for two hours. The final volume of the protease reaction was 120 µL. The reactions were stopped by the addition of 35 µL of concentrated sample buffer. For LC/E-16x concentration dependence, 10 µL of brain lysate were mixed with the indicated concentrations of protease and the final volume adjusted to 20 µL. The reaction was stopped by adding 60 µL of concentrated sample buffer.

### Neuronal cultures and intoxication assay

Primary cultures of rat cerebellar granule neurons (CGNs) were prepared as previously described (Azarnia Tehran and Pirazzini 2018). Briefly, cerebella were isolated from 6- to 8-day-old rats, mechanically disrupted and trypsinized in the presence of DNase I. Cells were then collected and plated into 24 well plates pre-coated with poly-L-lysine (50 µg/ml) at a cell density of 4 × 10^5^ cells per well. Cultures were maintained at 37 °C, 5% CO2, 95% humidity in BME (Basal Medium Eagle) supplemented with 10% fetal bovine serum, 25 mM KCl, 2 mM glutamine and 50 µg/ml gentamicin (hereafter indicated as complete culture medium). To arrest growth of non-neuronal cells, cytosine arabinoside (10 µM) was added to the complete culture medium 18 ± 24 h after plating.

CGNs at 6 to 8 days in vitro (DIV) were incubated with the indicated concentrations (from 5 pM to 70 nM) of the indicated BoNT/E or LC/E-wt-BoNT/Di or LC/E-16x-BoNT/Di in complete culture medium for 12–48 h at 37 °C. The specific proteolytic activity against PTEN and SNAP-25 was evaluated via immunoblotting with antibodies that recognize both the intact and the truncated form of the two proteins. Experiments were performed three times; immunoblots shown in the figures are representative of one of these experiments.

### Compound muscle action potential (CMAP)

Swiss-Webster female adult CD1 mice (24–26 g) were purchased from Charles River Laboratories Italia (Calco, Italy), fed with water and food ad libitum and maintained under 12-h light/12-h dark cycles. Animals were injected with the indicated toxins at the level of the hindlimb as previously described (Zanetti et al. 2017). One day after toxin injection, CMAPs were recorded from the gastrocnemius muscles of anesthetized mice following supramaximal stimulation with a 6002 Stimulator (Harvard Apparatus, Holliston, MA, USA) of the sciatic nerve (15 V, 4 ms, 0.5 Hz), approximately 0.5 mm below the sciatic notch, as previously described (Zanetti et al. 2019). Electromyographic signals were amplified using a EXT-02 F amplifier (NPI Electronic, Tamm, Germany) and digitized with a CW-INT-20USB interface (NPI Electronic). Data traces were captured with WinEDR and analysed using pClamp10.

### Immunoblotting

Cells were directly lysed with Laemmli sample buffer containing protease inhibitors (Roche). Cell lysates were loaded onto NuPage 4–12% Bis-Tris gels (Life technologies) and separated by electrophoresis in MOPS buffer (Life technologies). Proteins were transferred onto Protran nitrocellulose membranes (Whatman) and saturated for 1 h in PBS-T (PBS, 0.1% Tween 20) supplemented with 5% non-fatty milk. Incubation with primary antibodies for PTEN (138 G6, Cell Signaling Technology) and SNAP-25 (SMI-81, Abcam) were performed overnight at 4 °C. The membranes were then washed three times with PBS-T and incubated with appropriate secondary antibodies for 1 h. Membranes were washed three times with PBS and proteins revealed either with an Odyssey imaging system (LI-COR Bioscience) or with an Uvitec gel doc system (Uvitec Cambridge).

## Results

### Production and proteolytic activity of LC/E mutants

**Fig. 1:**
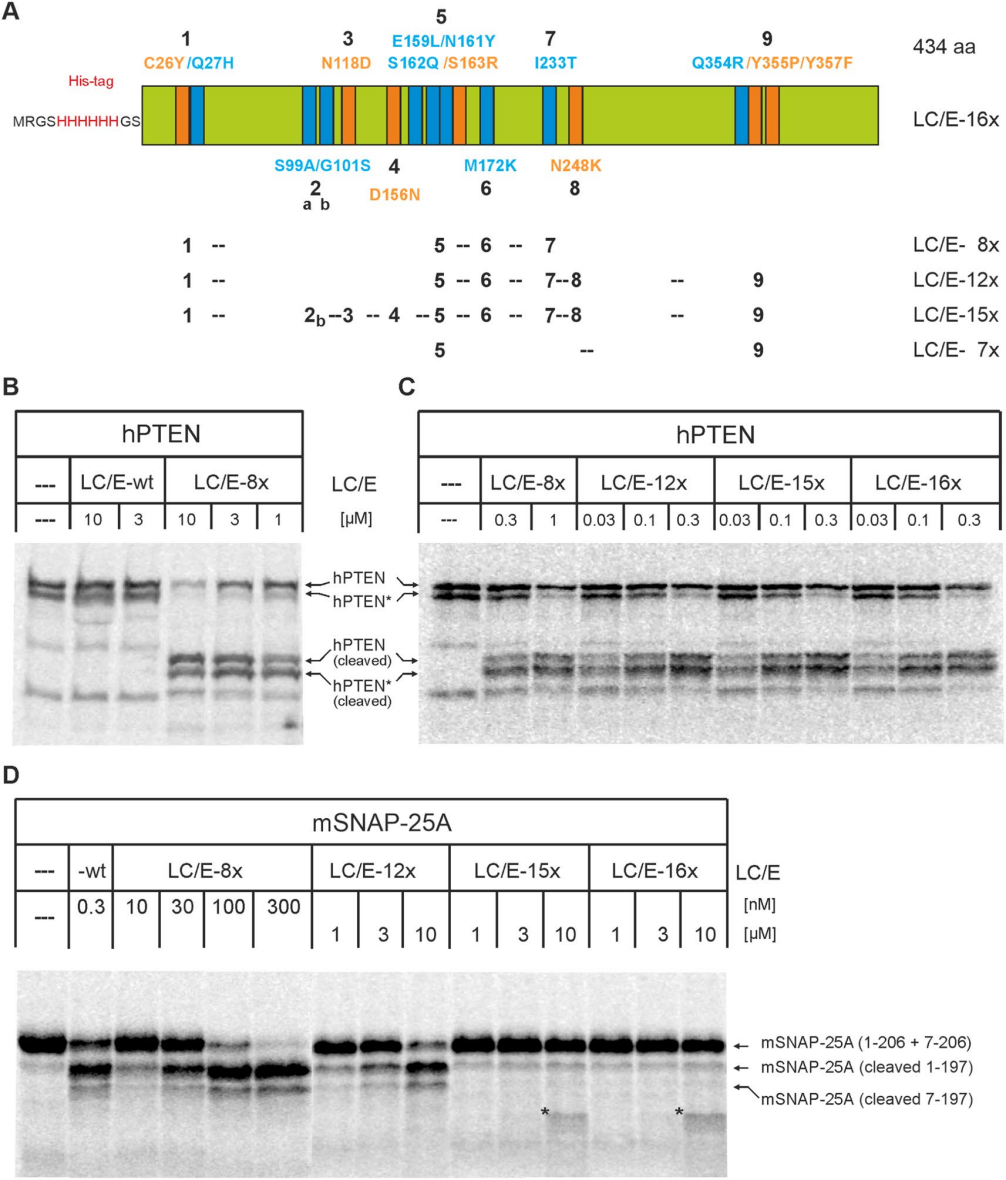
Activity of LC/E mutants on PTEN and SNAP-25. (**A**) The schematic of the PTEN adapted LC/E shows the 16 amino acid mutations in single letter code and their position within LC/E. Mutations colored blue resulted from positive selection for cleavage of PTEN, whereas those given in orange color resulted from negative selection for proteolysis of SNAP-25. Numbers below the schematic indicate the mutations or group of mutations assembled in produced interim LC/E mutants. E. g. LC/E-8x comprises group 1 (mutations C26Y, Q27H) plus group 5 (mutations E159L, N161Y, S162Q, S163R) plus M172K (6) plus I233T (7), whereas LC/E-15x comprises all but one mutation (S99A). (**B-D**) Cleavage of PTEN and SNAP-25 by LC/E mutants. Substrates were generated as ^35^S-labled proteins by in vitro transcription/translation and incubated in the presence of various concentrations of LC/E-wt or LC/E mutants for 1 h at 37 °C. Reactions were stopped by addition of pre-chilled SDS-PAGE sample buffer, boiled, and then run on 12.5% gels. After fixation of proteins, gels were dried and radioactively labeled proteins visualized by phosphorimaging. The asterisks mark an unidentified product

Recently a mutated LC/E was reported to cleave the phosphatase and tensin homolog (PTEN)(Blum et al. 2021), a tumor suppressor protein involved in cellular signaling (Duraikannu et al. 2019). However, this study did not investigate its activity as part of the full-length BoNT/E in a cell-based assay, requiring its translocation through the endosomal membrane after uptake in recycling vesicles. In addition, information about the individual contribution of mutations on cleavage of PTEN and the authentic substrate SNAP-25 is mainly confined to revertants of the 16-fold mutated LC. To fill this gap and to produce and characterize the 16-fold mutated full-length BoNT/E, we stepwise assembled the 16 mutations in an *E. coli* expression plasmid for LC/E (Fig. 1A).

The final 16-fold mutated LC/E expression plasmid was generated performing consecutive gene tailor reactions in which nearby mutations were stepwise combined (Fig. 1A). Thus, nine interim expression plasmids were also obtained. Of these, first the final mutant was produced and purified by means of its N-terminal hexahistidin tag together with LC/E-8x, -12x, -15x. All mutants provided yields exceeding 10 mg per liter of *E. coli* culture and remained soluble during dialysis.

The proteolytic activity of mutant LCs was tested in in vitro cleavage assays using ^35^S-methione labeled human PTEN and mouse SNAP-25A. Synthesis of PTEN led to two major protein products of ~ 47 kDa and ~ 43 kDa (Fig. 1B, C), the latter is probably due to initiation of translation at an in frame ATG codon 35 triplets downstream. As expected, LC/E-wt did not lead to a mobility shift of PTEN even when used at 10 µM final concentration, consistent with the lack of proteolytic activity. In contrast, a 10 µM concentration of LC/E-8x cleaved PTEN almost completely (Fig. 1B). In agreement with the scissile peptide bond location at position S302-I303 (Blum et al. 2021) both full-length and the N-terminally truncated PTEN were converted to faster migrating proteins. Their calculated molecular weights are 35.6 and 31.5 kDa, respectively. Their C-terminal cleavage product cannot be visualized as it lacks a methionine residue. Figure 1C shows that at 0.3 µM concentration, LC/E-8x leads to almost 50% cleavage of PTEN. Addition of mutation N248K and group 9 mutations (Q354R, Y355P, Y357F) leading to LC/E-12x, further increased the activity on PTEN (50% cleavage at ~ 200 nM; Table 1), whereas additional assembly of mutations D156N, N118D, G101S, and S99A in LC/E-12x did not lead to further increase of activity (LC/E-15x and LC/E-16x; Fig. 1C; Table 1).

**Table 1:** Activity of LC/E mutants to hPTEN in in vitro cleavage assays

	% cleavage^a^						
**LC/E**	**10 nM**	**30 nM**	**100 nM**	**300 nM**	**1000 nM**	**3000 nM**	**10,000 nM**
wt	n. a.^a^	n. a.	n. a.	n. a.	n. a. ^a^	0.4 ± 0.6 (2)^b^	0.5 ± 0.6 (2)
8x	1.3 ± 0.8 (2)	4.4 ± 2.6 (3)	13.6 ± 5.4 (3)	30.2 ± 8.7 (4)	51.5 ± 7.8 (5)	67.2 ± 4.5 (3)	83.4 ± 2.9 (2)
12x	7.1 ± 0.0 (2)	24.9 ± 4.1 (3)	36.8 ± 5.2 (3)	63.2 ± 3.0 (3)	76.1 ± 6.1 (3)	89.0 ± 0.0 (1)	n. d.
15x	15.4 ± 5.7 (2)	29.1 ± 2.9 (3)	46.9 ± 6.0 (3)	68.6 ± 3.6 (3)	80.3 ± 2.5 (2)	n. d.	n. d.
16x	15.7 ± 6.5 (2)	26.5 ± 2.4 (3)	43.6 ± 10.3 (5)	62.1 ± 7.1 (5)	84.1 ± 2.9 (4)	n. d.	n. d.
7x	n. d.	n. d.	1.5 ± 0.9 (3)	4.8 ± 1.7 (3)	17.7 ± 7.4 (4)	41.5 ± 5.7 (4)	60.4 ± 4.1 (4)
7x-159	n. d.	n. d.	n. d.	n. d.	3.2 ± 1.6 (2)	7.7 ± 1.9 (3)	16.8 ± 3.7 (3)
7x-162	n. d.	n. d.	n. d.	n. d.	0.1 ± 0.5 (2)	3.0 ± 1.0 (3)	8.5 ± 2.0 (3)
7x-59/62	n. d.	n. d.	n. d.	n. d.	n. d.	0.1 ± 0.4 (2)	0.5 ± 0.6 (2)
7x-161	n. d.	n. d.	n. d.	n. d.	6.9 ± 0.9 (3)	16.0 ± 5.3 (3)	29.2 ± 6.5 (3)
7x-163	n. d.	n. d.	n. d.	n. d.	12.2 ± 0.7 (3)	26.0 ± 5.5 (3)	38.8 ± 3.5 (3)
7x-61/63	n. d.	n. d.	n. d.	n. d.	1.0 ± 0.3 (3)	2.1 ± 0.3 (3)	6.3 ± 0.6 (3)
mut 5	n. d.	n. d.	n. d.	35.3 ± 0.0 (1)	40.9 ± 2.1 (2)	54.0 ± 13.7 (2)	64.2 ± 12.2 (2)
mut 9	n. d.	n. d.	n. d.	n. d.	1.4 ± 1.1 (2)	3.1 ± 1.6 (2)	2.2 ± 0.1 (2)
16x-159	n. d.	n. d.	5.3 ± 1.4 (3)	13.4 ± 2.6 (3)	28.2 ± 9.7 (2)	41.9 ± 0.0 (1)	59.4 ± 0.0 (1)
16x-162	n. d.	n. d.	4.2 ± 3.9 (3)	5.1 ± 3.8 (2)	11.4 ± 0.4 (2)	33.3 ± 0.0 (1)	51.3 ± 0.0 (1)
16x-59/62	n. d.	n. d.	0.8 ± 0.2 (2)	0.2 ± 0.0 (2)	1.5 ± 0.5 (3)	5.5 ± 0.0 (1)	14.9 ± 0.0 (1)

^a^Cleavage assays were conducted at various LC concentrations using [^35^S]-methionine labeled hPTEN as substrate for 1 h at 37 °C in toxin assay buffer. Percentage of cleavage was calculated after phosphorimaging as the ratio between radioactivity of cleaved PTEN to intact plus cleaved PTEN

^b^ numbers in brackets specify number of experiments. n.d., not determined

In vitro transcription/translation of SNAP-25A resulted in a major product of a calculated mass of 23.4 kDa and a minor one running approximately 1 kDa longer, probably as result of translational start at Met-codon in position 7 (Fig. 1D). LC/E-wt gives rise to more than 50% cleavage of SNAP-25 when applied at 0.3 nM concentration, in agreement with previously published data (Vaidyanathan et al. 1999). The resulting cleavage products comprise 180 and 26 amino acids. The latter, the C-terminal product, could not be resolved. Similar extent of SNAP-25A proteolysis required about 1000-fold higher LC/E-8x concentration, and was achieved with LC/E-12x only when increasing the concentration to more than 3 µM (Fig. 1D; Table 2). SNAP-25A appeared refractory to LC/E-15x and LC/E-16x. Reliable cleavage was not seen at the highest applied concentration of 10 µM (Fig. 1D; Table 2).

**Table 2:** Activity of LC/E mutants to in vitro translated mSNAP-25 in in vitro cleavage assays

	% cleavage^a^						
**LC/E**	**10 nM**	**30 nM**	**100 nM**	**300 nM**	**1000 nM**	**3000 nM**	**10,000 nM**
8x	12.3 ± 4.6 (3)	33.8 ± 2.1 (3)	64.6 ± 9.9 (3)	86.5 ± 2.2 (3)	90.2 ± 0.0 (1)	n. d.	n. d.
12x	n. d.	n. d.	n. d.	3.5 ± 1.2 (3)	7.0 ± 1.5 (5)	22.0 ± 4.9 (5)	75.9 ± 10.4 (5)
15x	n. d.	n. d.	n. d.	n. d.	0.7 ± 0.6 (3)	1.3 ± 0.3 (3)	4.0 ± 1.5 (3)
16x	n. d.	n. d.	n. d.	n. d.	1.0 ± 0.9 (3)	1.1 ± 0.2 (3)	4.1 ± 1.3 (3)
7x	20.6 ± 9.1 (4)	47.9 ± 17.6 (4)	84.5 ± 7.6 (5)	92.0 ± 2.1 (4)	94.7 ± 0.0 (1)	n. d.	n. d.
	**0.1 nM**	**0.3 nM**	**1 nM**	**3 nM**	**10 nM**	**30 nM**	**100 nM**
wt	66.8 ± 8.6 (3)	85.1 ± 18.7 (4)	94.2 ± 2.7 (4)	94.7 ± 0.0 (1)	n. d.	n. d.	n. d.
7x	n. d.	1.2 ± 0.0 (1)	1.3 ± 2.3 (2)	12.8 ± 10.4 (3)	see above	see above	see above
7x-159	2.6 ± 0.0 (1)	6.4 ± 2.8 (4)	14.1 ± 2.9 (4)	38.2 ± 7.6 (4)	69.6 ± 6.5 (3)	91.9 ± 1.8 (3)	n. d.
7x-162	n. d.	-0.4 ± 0.0 (1)	0.1 ± 1.1 (2)	-0.2 ± 1.0 (2)	-0.5 ± 0.5 (2)	0.4 ± 0.1 (2)	1.8 ± 0.0 (1)
7x-59/62	n. d.	n. d.	n. d.	3.1 ± 0.0 (1)	4.6 ± 4.0 (2)	24.5 ± 5.2 (2)	78.0 ± 4.4 (2)
7x-161	n. d.	n. d.	1.8 ± 0.1 (2)	3.9 ± 1.2 (3)	9.3 ± 8.8 (4)	28.9 ± 20.6 (4)	63.1 ± 19.8 (4)
7x-163	20.0 ± 7.6 (2)	50.5 ± 3.9 (2)	91.7 ± 1.6 (3)	95.5 ± 2.1 (2)	n. d.	n. d.	n. d.
7x-61/63	2.9 ± 0.8 (2)	9.6 ± 2.5 (2)	24.5 ± 5.7 (3)	71.1 ± 1.3 (3)	92.2 ± 4.9 (2)	92.2 (1)	n. d.
mut 5	3.7 ± 0.0 (1)	15.8 ± 0.0 (1)	62.2 ± 0.0 (1)	91.9 ± 0.0 (1)	n. d.	n. d.	n. d.
mut 9	1.7 ± 0.0 (1)	5.3 ± 0.0 (1)	31.4 ± 0.0 (1)	54.6 ± 0.0 (1)	n. d.	n. d.	n. d.

^a^Cleavage assays were conducted at various LC concentrations using [^35^S]-methionine labeled mSNAP-25 as substrate for 1 h at 37 °C in toxin assay buffer. Percentage of cleavage was calculated after phosphorimaging as the ratio between radioactivity of cleaved SNAP-25 to intact plus cleaved SNAP-25

^b^ numbers in brackets specify number of experiments. n. d., not determined

Altogether, these data suggest that mutations within LC/E-8x and LC/E-12x are necessary to enable and enhance, respectively, the proteolytic activity of LC/E on PTEN, while the amino acid mutations leading to LC/E-15x are needed to turn off SNAP-25 cleavage, in keeping with the mutations identified by applying positive and negative selection constrains (Blum et al. 2021).

### Expression of PTEN-specific full-length BoNT/E mutants

**Fig. 2:**
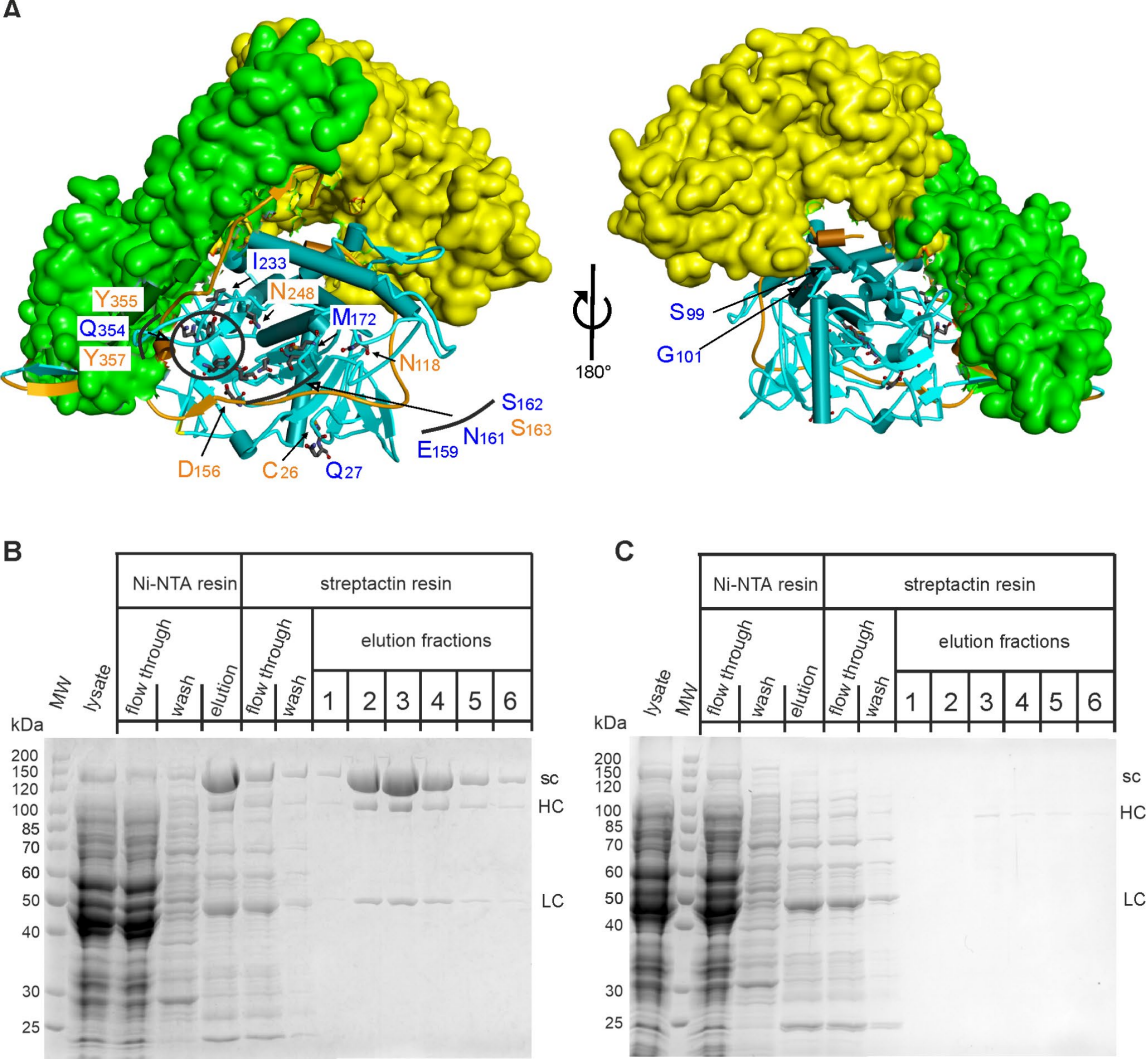
Production of full-length BoNT/E-16x. (**A**) Structure of BoNT/E (PDB ID: 3FFZ; (Kumaran et al. 2009)). The H_N_ (green) and H_C_ (yellow) domains are depicted as surface plot, whereas the belt region (brownish color) and LC (blue) are shown as ribbon diagram. Positions of amino acids which are mutated in BoNT/E-16x are indicated. Blue color specifies amino acids obtained by positive selection for PTEN cleavage. Orange color was used for amino acids identified by negative selection for SNAP-25 cleavage. Production of BoNT/E-wt (**B**) and BoNT/E-16x (**C**) was induced in *E. coli* M15pREP4 by means of pQE derived plasmids and IPTG during 15 h at 21 °C. Bacterial lysate was cleared by centrifugation and run through a Ni-NTA column. The imidazole eluate was further purified on a streptactin column. Samples of each purification step were analyzed by SDS-PAGE using 10% gels. Aliquots of 7.5 µl of streptactin elutions fractions were loaded. sc: single chain, HC: heavy chain, LC: light chain. Note that BoNT/E-16x could not be detected and that due to proteolytic activation by *E. coli* proteases a fraction of BoNT/E-wt splits up into HC and LC

The LC encoding part of the 16-fold mutated LC expression plasmid was used to replace the corresponding part of the full-length expression vector of BoNT/E (pH6BoNTEs-thro, (Rummel et al. 2009). pH6BoNTEs-thro-16x and the parental wild-type encoding plasmid were applied to produce the full-length proteins in order to characterize them in neuronal cultures. Unexpectedly, while the production and purification of BoNT/E-wt yielded protein in line with previous preparations in our lab (1–2 mg/L of *E. coli* culture) (Fig. 2A and B), no BoNT/E-16x at all appeared in the imidazole elution fraction of the Ni-NTA column and consequently in the eluates of the streptactin column (Fig. 2A, C). Re-analysis of pH6BoNTEs-thro-16x nucleotide sequence did not reveal any unwanted mutation and expression of another pH6BoNTEs-thro-16x clone proved also unsuccessful (not shown). Thus, a solubility issue seemed to be the likely explanation for the failure in purifying BoNT/E-16x.

**Fig. 3:**
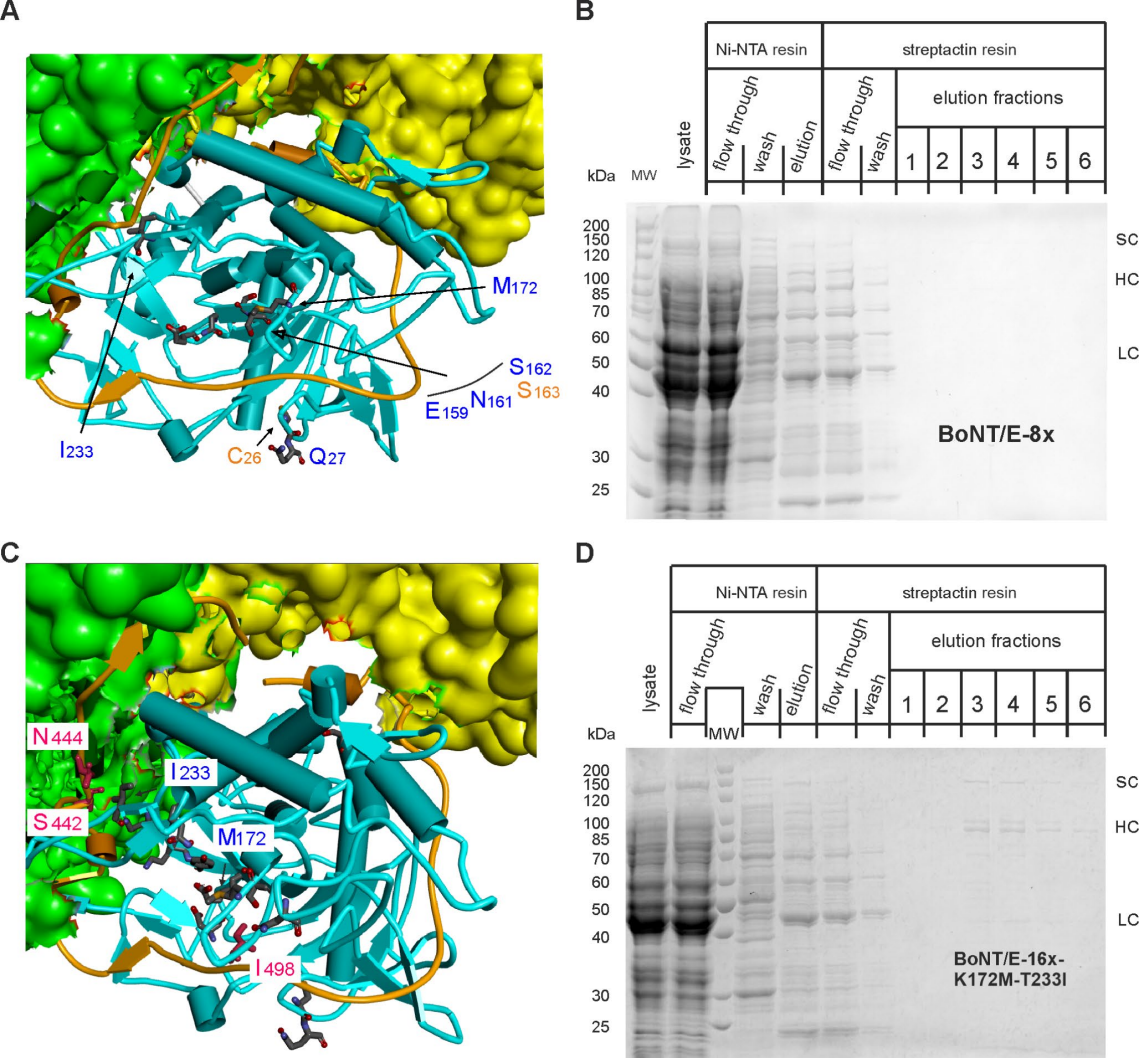
Production of full-length BoNT/E-8x and − 16x-K172M-T233I. (**A**,** C**) Close-up views of the LC/E (blue) and belt region (brownish color) structure depicted as ribbon diagram. Parts of the H_N_ (green) and H_C_ (yellow) domains are shown as surface plot. (**A**) Amino acids mutated in BoNT/E-8x are indicated and shown in stick representation. (**C**) Mutated amino acids in the LC of BoNT/E-16x are shown in stick representation and those reverted to the original residues are indicated in blue letters. Belt region amino acids in the neighborhood of the two reverted amino acids are represented in stick model (red) and specified. (**B**, **D**) Production of BoNT/E-8x and − 16x-K172M/T233I was induced in *E. coli* M15pREP4 by means of pQE derived plasmids during 15 h at 21 °C and proteins were purified and analyzed as detailed in Fig. 2. Aliquots of 7.5 µl of streptactin elutions fractions were loaded. sc: single chain, HC: heavy chain, LC: light chain. Note that BoNT/E-8x could not be detected, whereas a marginal amount of BoNT/E-16x-K172M/T233I was obtained compared to BoNT/E-wt (cp. Figure 2)

Interference of specific LC mutations with important interactions between LC and HC belt region might induce destabilization of the overall structure (Brunger et al. 2007). To shed light on this hypothesis, we first analyzed production of the intermediate BoNT/E-8x mutant, which bears amino acid replacement groups 1 and 5 and two mutations (I233T and M172K, respectively group 6 and 7) in close proximity to belt region residues (S442, N444, and I498, respectively) (Fig. 3A, C). Interestingly, pH6BoNTEs-thro-8x also failed to provide soluble BoNT/E (Fig. 3B), restricting the responsible candidates of the solubility issue to amino acids of groups 1 (C26Y and Q27H) or 5 (E159L, N161Y, S162Q and S163R), or replacements M172K or I223T (cp. Figure 1A). Second, as M172K or I233T are part of the LC-HC belt interface (Fig. 3C), we produced the BoNT/E-16x-mutant reverting these two mutations to the original amino acids. However, the resulting 14-fold mutant was merely obtained at a marginal scale (Fig. 3D).

**Fig. 4:**
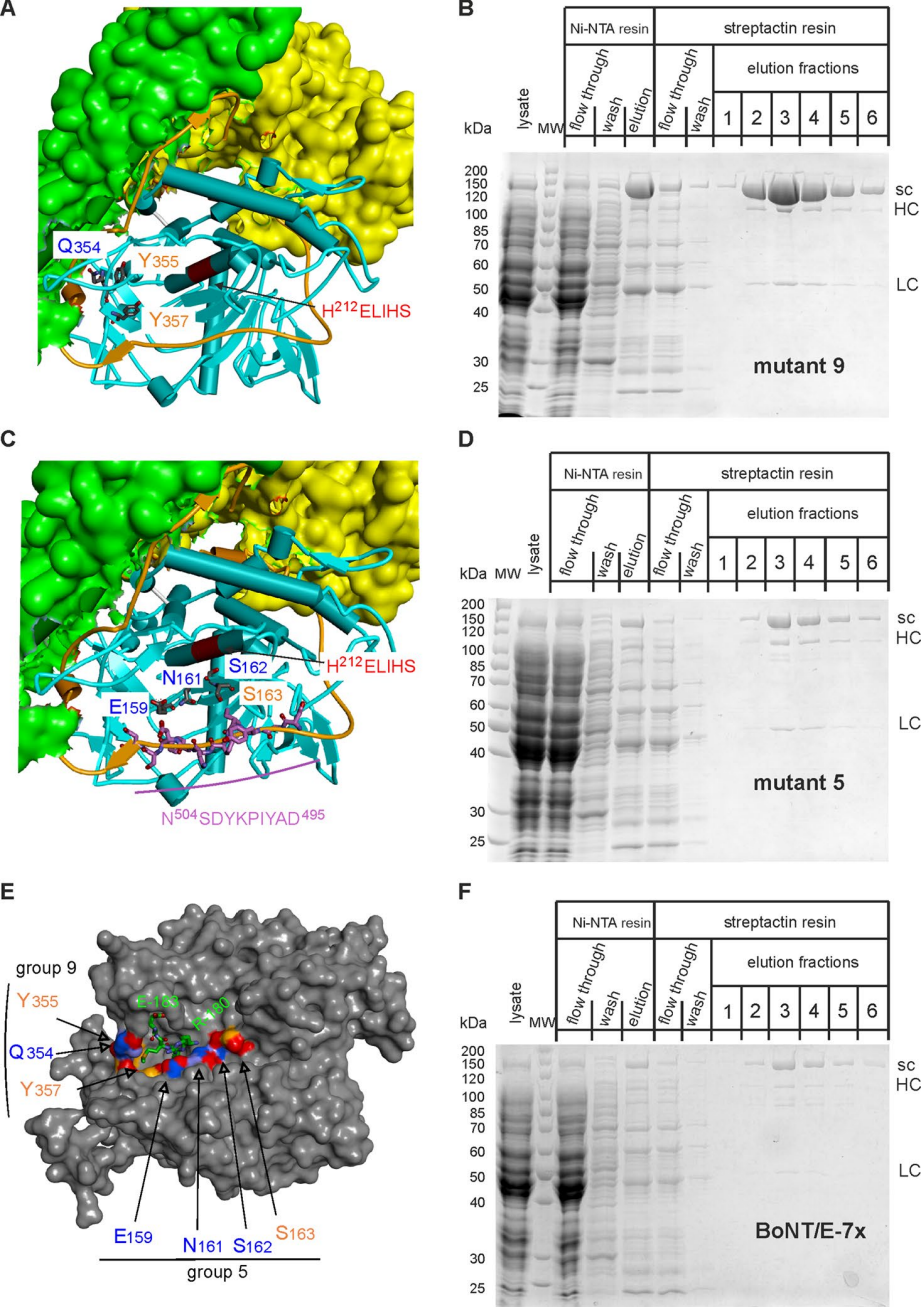
Production of various full-length BoNT/E mutants. (**A**,** C**) Close-up views of the LC/E (blue) and belt region (brownish color) structure depicted as ribbon diagram. Parts of H_N_ (green) and H_C_ (yellow) domains are shown as surface plot. Amino acids mutated in mutant 9 and mutant 5 are shown in stick model and indicated. The active center of BoNT/E is highlighted red and amino acids are specified. Belt region residues in the neighborhood of amino acids mutated in mutant 5 are presented in stick model (purple) and listed. (**E**) Surface plot of LC/E bound to a SNAP-25 derived tetramer peptide (PDB ID: 3D3X; (Agarwal and Swaminathan 2008)). Amino acids mutated in BoNT/E-7x are colored and specified. The SNAP-25 derived peptide R180-I-M-E183 comprising the scissile peptide bond R180-Ile-181 is shown in greenish color in the stick model. (**B**, **D**, **F**) BoNT/E-mutants were produced in *E. coli* M15pREP4 by means of pQE derived plasmids and IPTG during 15 h at 21 °C. Proteins were purified and analyzed as detailed in Fig. 2. Aliquots of 7.5 µl of streptactin elutions fractions were loaded. sc: single chain, HC: heavy chain, LC: light chain. Note that due to proteolytic activation by *E. coli* proteases fractions of BoNT/E preparations split up into HC and LC

Next, we focused on group 5 mutations, which are important for PTEN cleavage. These amino acids are part of the active site cavity surface and may interact with belt region amino acids due to spatial proximity (Fig. 4C, E). Additionally, we examined group 9 mutations (Fig. 4A), which are critical for abrogating SNAP-25 specificity (cp. Figure 1). The yield of full-length BoNT/E carrying the group 9 mutations was nearly identical to that of BoNT/E-wt (Fig. 4B), whereas the presence of group 5 mutations resulted in a drastic reduction in the yield of soluble protein (Fig. 4D). Consistently, the yield for the BoNT/E-7x mutant with the four group 5 mutations plus the three group 9 mutations was comparable to that of the group 5 BoNT/E-mutant (Fig. 4E, F). Altogether, these results indicate that the group 5 mutations are the major cause of the solubility issue observed in the final 16-fold mutated BoNT/E.

### Dissection of the effect of individual group 5 mutations on PTEN cleavage

**Fig. 5:**
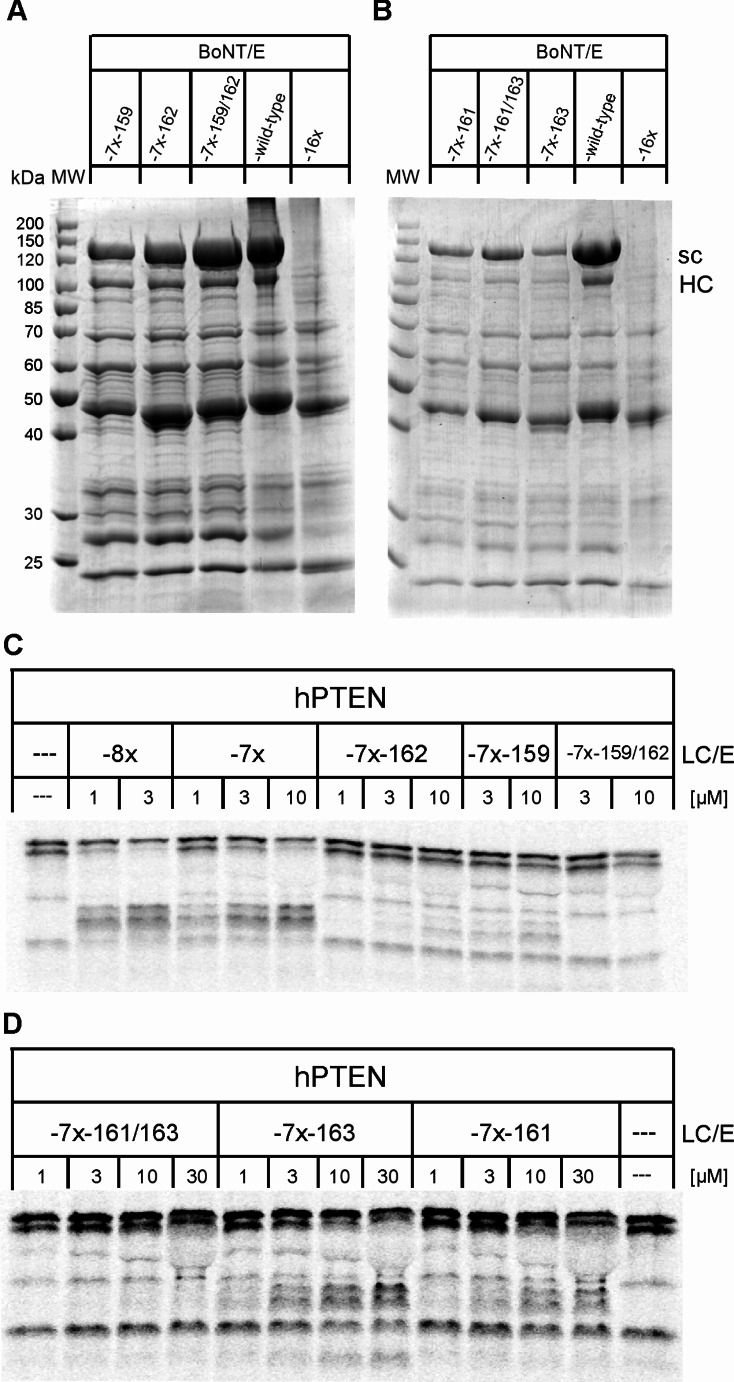
Characterization of group 5 revertants. (**A**, **B**) Full-length BoNT/E group 5 revertants were produced and purified as detailed in the legend to Fig. 2. Aliquots of 7.5 µl of streptactin elutions fractions of each mutant were analyzed by SDS-PAGE using 10% gels and compared with those of BoNT/E-wild-type and BoNT/E-16x. (**C**, **D**) The activity of LC/E group 5 revertants was assessed on PTEN as described in the legend to Fig. 1. Various concentrations of revertants in positions 159 and 162 (**C**) or positions 161 and 163 (**D**) as well as of LC/E-8x and LC/E-7x for comparison were applied. Samples were run on 12.5% gels and radiolabeled proteins were finally visualized by phosphorimaging

Production of full-length BoNT/E lacking group 5 mutations would be the corollary of the results of the protein production analyses. However, group 5 mutations are vitally important for the proteolytic activity on PTEN (cp. Figure 1). Therefore, we studied the individual impact of these mutations on both solubility and PTEN cleavage. All revertant mutations, L159E, Y161N, Q162S and R163S, as well as L159E/Q162S and Y161N/R163S, were generated in the background of BoNT/E-7x or LC/E-7x (cp. Fig, 1), respectively, as this septuple mutant could be produced as full-length BoNT/E, although with a low yield (Fig. 4F). The reversal of the group 5 mutations resulted in quite different effects on the yield of soluble BoNT/E, which increased in the order R163S, Y161N, L159E, Q162S. Combining the latter two revertants (resulting in the quintuple mutant BoNT/E-N161Y/S163R/Q354R/Y355P/Y357F) restored the expression level to that of wild-type BoNT/E (Fig. 5A). The combination of revertants Y161N and R163S (i.e. BoNT/E-E159L/S162Q/Q354R/Y355P/Y357F) improved the yield of BoNT/E, although the effect was considerably less pronounced (Fig. 5B).

Next, we studied the effect on PTEN cleavage of the corresponding reverse mutations in LC/E-7x. Among the single revertants, the PTEN cleavage activity decreased in the order R163S, Y161N, L159E, Q162S (Fig. 5C, D; Table 1). LC/E-7x-159-162, which combines the two most impactful reversions, displayed no proteolytic activity (Fig. 5C; Table 1). To validate these findings, the reversion of E159L and S162Q was also assessed in the background of the 16-fold mutated LC/E. LC/E-16x-Q162S and LC/E-16x-L159E exhibited approximately 30-fold and 50-fold reduction in activity toward PTEN compared to LC/E-16x. Furthermore, LC/E-16x-L159E/Q162S displayed minimal activity, even at a 10 µM concentration (Table 1), highlighting the critical role of these two mutations for PTEN cleavage. In conclusion, the mutations that most negatively affect the yield of soluble BoNT/E, E159L and S162Q, are also the most crucial for effective PTEN cleavage. Thus, the fact that a significant gain in PTEN cleavage activity is inextricably linked to a major reduction in solubility seems to be an unresolvable problem.

### Production and testing on cultured cortical neurons of LC/E-BoNT/Di fusion proteins

**Fig. 6:**
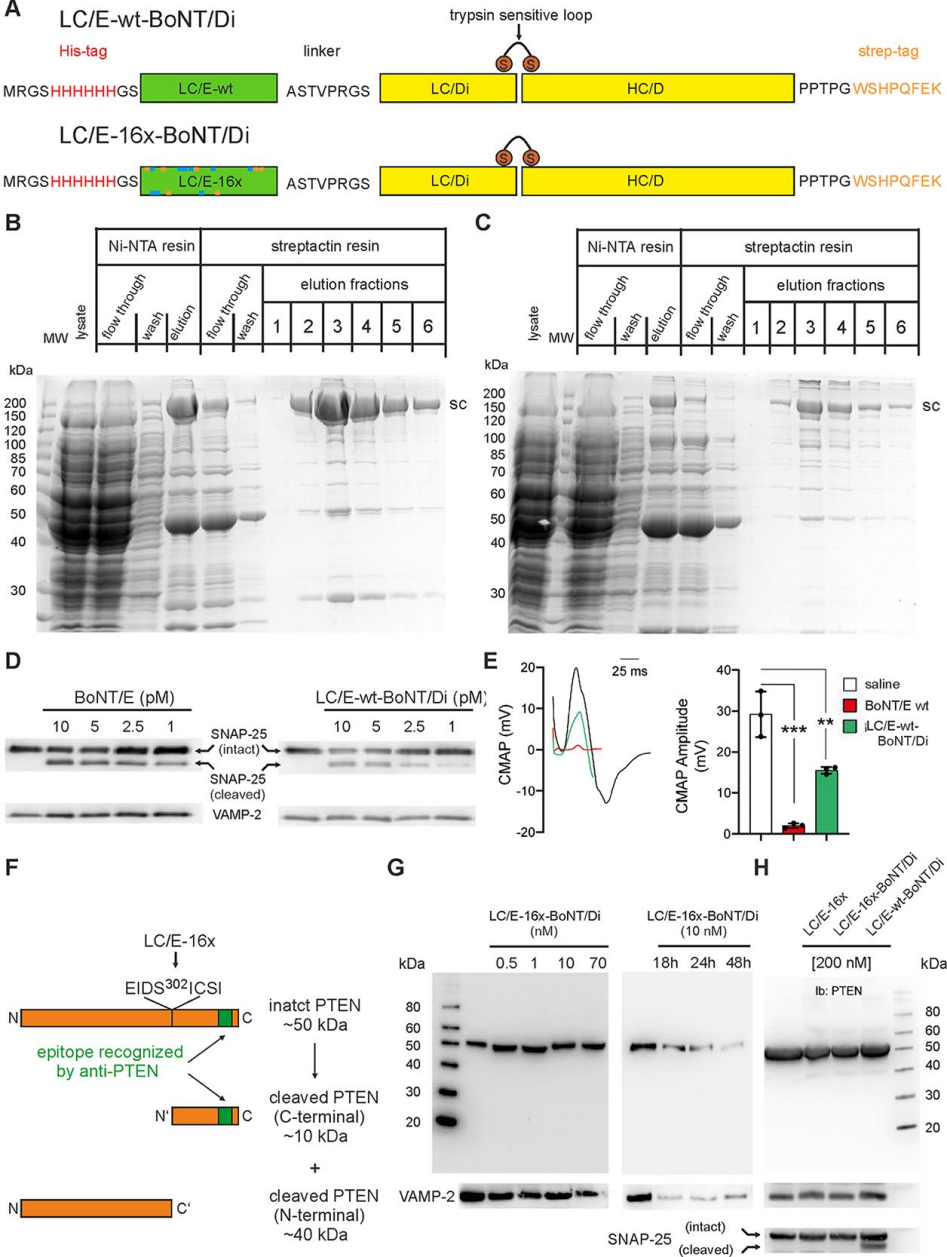
Production and activity in primary rat cerebellar granule neurons of LC/E-wt-BoNT/Di and LC/E-16x-BoNT/Di. (**A**) Schematic illustrating the structure of LC/E-BoNT/Di fusion proteins. (**B**, **C**) LC/E-wt- and LC/E-16x-BoNT/Di fusion proteins were produced in *E. coli* M15pREP4. Proteins were purified and analyzed as stated in the legend to Fig. 2. (**D**) Cultured rat CGNs were treated with BoNT/E or LC/E-wt-BoNT/Di fusion proteins for 12 h at the indicated concentrations and subsequently lyzed directly in sample buffer. Samples were heat denatured and proteins separated by 4–12% SDS-PAGE (NuPAGE). SNAP-25, and VAMP-2 were detected using specific antibodies by Western blotting. Immunoblots shown are representative of three experiments performed. (**E**) Representative single traces and amplitude quantification of CMAP recordings in the gastrocnemius muscles elicited by supramaximal sciatic nerve stimulation (0.5 Hz) 24 h after injection of vehicle (black), BoNT/E (red) or LC/E-wt-BoNT/Di (green). Quantification represents the mean value obtained from 10 CMAP evoked in three individual animals. Statistical analysis was performed by t-test with Welch correction, ** p-value: 0.01 and *** p-value: 0.001. (**F**) Schematic illustrating the structure of PTEN. The cleavage site amino acids for LC/E-16x are depicted and the region recognized by the used PTEN specific monoclonal antibody is highlighted green. (**G**) Cultured CGNs were treated with the indicated concentrations of LC/E-wt-BoNT/Di diluted in complete culture medium and added to the neurons for 12 h (left panel) or for 18, 24, 48 h (right panel). Toxin activity was evaluated by monitoring whole and truncated PTEN immunoreactivity on the entire Western blot membrane. After revealing PTEN, the membranes were stripped and VAMP-2 immunoreactivity was assessed to evaluate the total protein content. Immunoblots shown are representative of three experiments performed. (**H**) A brain lysate was treated for 2 h at 37 °C with the indicated protease used at a concentration of 200 nM, representing the highest achievable according to the yield of LC/E-wt-BoNT/Di production. The reaction was stopped by adding sample buffer directly into the test tube. Toxin activity was evaluated by monitoring whole and truncated PTEN immunoreactivity on the entire Western blot membrane. After revealing PTEN, the membranes were stripped and SNAP-25 and VAMP-2 immunoreactivity were examined as internal controls for the cleavage assay and protein content, respectively. Immunoblots shown are representative of three experiments performed

To overcome the problem of defective interactions between the mutated LC/E and HC/E belt region, we reclaimed a previously successful BoNT fusion protein strategy. In this approach, LC/E-16x was fused to the N-terminus of a proteolytically inactive full-length BoNT/D generating LC/E-16x-BoNT/Di (Bade et al. 2004). The goal was to link the LC/E-16x protease, deprived of belt encirclement, to the LC/D protease, thus harnessing the BoNT/D molecule as a vehicle. This design aimed to preserve the solubility of LC/E-16x while maintaining the highly specific neuronal binding and delivery of the cargo protein to the neuronal cytosol (Fig. 6A). As a control, an expression plasmid for LC/E-wt-BoNT/Di was also designed. Figure 6B and C show that this strategy allowed the production of both LC/E-wt-BoNT/Di and LC/E-16x-BoNT/Di. Although the latter was obtained in a lower amount, both fusion proteins were produced in sufficient quantities for testing their activities on primary neuronal cultures. For this purpose, we used cerebellar granule neurons, which are highly susceptible to BoNTs and allow a simple assessment of their proteolytic activity by Western blotting (Azarnia Tehran and Pirazzini 2018).

When added to the culture medium of CGNs for 12 h, LC/E-wt-BoNT/Di successfully entered the neurons and cleaved SNAP-25 at levels comparable to those achieved with wt-BoNT/E (Fig. 6D), as detected with an antibody able to recognize both the intact and the cleaved form of the protein. Consistently, LC/E-wt-BoNT/Di also blocked neurotransmission in the gastrocnemius muscle, as demonstrated by a reduction in the compound muscle action potential one day after local toxin injection into the hind limb of mice. In this case, however, the effect was less than that of wt-BoNT/E (Fig. 6E).

Building on previous results showing that BoNT/Di could effectively deliver the cargo LC/E into the neuronal cytosol, mirroring earlier findings with LC/A in both cultured neurons and at the neuromuscular junction (Bade et al. 2004), we next tested whether LC/E-16x-BoNT/Di could deliver into CGNs also the PTEN-specific protease. Given the unpredictable susceptibility of CGNs to PTEN cleavage, neurons were treated with a broad range of LC/E-16x-BoNT/Di concentrations, from 0.5 to 70 nM. The incubation time was 12 h, a duration sufficient for LC/E to produce detectable SNAP-25 cleavage at low pM concentration, and PTEN cleavage was monitored with a monoclonal antibody targeting a C-terminal epitope. Although the immunogenic sequence was not reported by the manufacturer, a detailed study comparing the reactivity of various anti-PTEN antibodies mapped this epitope at the extreme C-terminus of the protein (Mingo et al. 2019), downstream of the LC/E-16x cleavage site (Fig. 6F). Consequently, the expected Western blotting pattern following PTEN cleavage would show a reduction or disappearance of the band corresponding to intact PTEN accompanied by the emergence of a new lower band, approximately 10 kDa in size, corresponding to the PTEN fragment generated by LC/E-16x cleavage and containing the epitope recognized by the antibody. As shown in Fig. 6G, treatment of CGNs with LC/E-16x-BoNT/Di, used at about 100-fold higher concentration than LC/E-wt-BoNT/Di, did not result in a detectable decrease of the intact PTEN band nor did it cause the appearance of the 10 kDa fragment, indicating no cleavage of the protein. To assess whether a longer incubation period might enhance PTEN cleavage, we extended the treatment to 48 h. While this resulted in a slight reduction of the intact PTEN band compared to 12 h and control, the 10 kDa cleaved fragment did not appear. Rather, we observed a reduction in SNARE protein levels, possibly indicating some cytotoxicity of LC/E-16x-BoNT/Di.

Overall, these results indicate that LC/E-16x-BoNT/Di failed to hydrolyze PTEN when added exogenously to the neuronal culture. The lack of PTEN cleavage could be due to several factors (see discussion), including an insufficient delivery of active LC/E-16x in the cytosol. To test this possibility, we added LC/E-16x and LC/E-16x-BoNT/Di directly to a neuronal lysate, bypassing the cell entry step to allow direct interaction between the LC/E-16x protease and neuronal PTEN. The concentration of LC/E-16x and LC/E-16x-BoNT/Di used was 200 nM, i.e., the maximum achievable based on the production yield of LC/E-16x-BoNT/Di. As shown in Fig. 6H, neither LC/E-16x and LC/E-16x-BoNT/Di cleaved PTEN under these conditions. However, the same experiment with LC/E-wt-BoNT/Di yielded a clear signal of cleaved SNAP-25, confirming that the experimental conditions were appropriate for proteolytic activity. Since the concentration of LC/E-16x-BoNT/Di may have been represented a limiting factor in this experiment, we repeated the experiment with the isolated LC/E-16x protease to test whether neuronal PTEN could be cleaved at higher concentrations. This allowed for LC/E-16x concentrations in the brain lysate of up to 3000 nM. Nonetheless, no PTEN cleavage was observed across the 300–3000 nM range (data not shown).

## Discussion

BoNT/A and BoNT/B are used for decades as effective pharmaceuticals for diseases that rely on overshooting neurotransmitter release. Recent research activities aimed at adapting the enzymatic activity of BoNTs to different intracellular targets in order to enlarge the range of use to further diseases (Blum et al. 2021; Dyer et al. 2022; Sikorra et al. 2020). The initial goal of this study was to examine whether the recently reported PTEN specific LC/E might serve as a potential strategy to support axonal regeneration after injury and neuronal survival in neuromuscular disorder. Leveraging the exceptional neurospecificity of BoNTs would have the advantage to target a PTEN-specific protease specifically in mature neurons while preserving PTEN function in other cell types, including neural stem cells and progenitors, oligodendrocytes, astrocytes and Schwann cells. This selective targeting would minimize the risk of developing primary nervous system tumors, such as glioblastomas, schwannomas, neurofibromas, or malignant peripheral nerve sheath tumors. Likewise, the transient and reversible nature of BoNT protease activity would make neoplastic transformation in neuronal cells unlikely, limiting the risk of developing tumors such as neuroblastomas, which are however extremely rare neurodevelopmental tumors in adults (Maris 2010).

Blum and colleagues (Blum et al. 2021) demonstrated partial PTEN and lack of SNAP-25 cleavage in cultured primary neurons upon LC/E-16x transduction with a lentiviral system. Here, we wanted to generate the full-length BoNT/E molecule bearing the 16-fold mutated LC/E in order to study the properties of this molecule under physiologically meaningful conditions. However, production in *E. coli* failed. General failure of full-length BoNT/E production could be ruled out as wild-type BoNT/E (as well as several interstage mutants) could be purified in decent amounts. In fact, we deem intrinsic structural instability caused by one or more of the mutations as the reason for solubility issues. Production of BoNT/E-8x (Fig. 3B) also failed as well as BoNT/E-16x in which M172K and I233T were reverted to original amino acids (Fig. 3D). This leaves only group 1 and group 5 mutations (Fig. 1) as sources of solubility problems. Among them C26Y and Q27H are unlikely to affect BoNT/E solubility as cysteine-26 and glutamine-27 are located remote to belt region, H_N_, and H_C_ (Fig. 3A). In addition, both amino acids are solvent exposed and not involved in intra LC interactions as underscored by the similar production yield of wild-type and 16-fold mutated LC/E. The side chains of group 5 amino acids 159, 161 and 163 line up face to face with segment D-495 to N-504 of the H_N_ belt region (Fig. 4C). Consequently, mutation of Glu-159 to leucine, of Asn-161 to tyrosine, and of Ser-163 to arginine, might eliminate important H-bond interactions with Tyr-501 and for the latter two with Asp-502, whose elimination may lead to structural instability. Consistently, each reversion mutation at these positions resulted in improved production yield. However, why the strongest effect on the restoration of the production yield of full-length BoNT/E was achieved by reverting the mutation in position 162 (Gln to Ser) is unclear. The hydroxymethyl side chain of serine points towards the catalytic center, i.e., residues Glu-213 and Ser-217, and thus could only be implicated in intra LC interactions. Production of BoNT/E lacking the four group 5 mutations was not tested, as they are key to PTEN cleavage (see the following section). Reversion mutations of each of the four amino acids significantly reduced the PTEN cleavage capability disclosing the mutation at position 162 as the most important one (Table 1). This agrees with published results for reversion mutants analyzed by luciferase-based activity assays (Blum et al. 2021). Therefore, none of the four group 5 amino acid mutations is expendable with regard to PTEN cleavage efficiency, but each one contributes to the solubility issue of full-length BoNT/E-16x.

Our solution to this seemingly unsolvable problem was to deliver PTEN cleaving 16-fold mutated LC/E as cargo protein of a proteolytically inactive BoNT. The fusion proteins LC/E-wt-BoNT/Di and LC/E-16x-BoNT/Di were both expressed and purified in amounts suitable for their testing in primary neurons, suggesting that the solubility issue of the LC/E-16x could be overcome through this strategy. LC/E-wt-BoNT/Di caused SNAP-25 cleavage at levels similar to those produced by a recombinant wild-type BoNT/E, confirming that the BoNT/Di framework was able to deliver the cargo protease in an active state. Unexpectedly, LC/E-16x-BoNT/Di was unable to yield any detectable cleavage product of PTEN, although used at reasonably higher concentration than LC/E-wt-BoNT/Di and BoNT/E.

Possible reasons for the absence of detectable PTEN proteolysis products could be due to factors ascribable to its cellular biology: (i) the scissile peptide bond of PTEN is inaccessible for LC/E-16x due to complex formation with other proteins; (ii) generated cleavage products of PTEN are readily depleted by the intracellular protein degradation machinery; (iii) the cleaved PTEN is readily replaced with fresh PTEN. The possibility of a shielded PTEN scissile bond can be addressed in future experiments by applying LC/E-16x-BoNT/Di to different neuronal cultures or to CGNs under different intracellular conditions after external stimuli. Transient presence of PTEN cleavage products could be analyzed by blocking proteasomal degradation by inhibitors such as MG-132. Alternatively, the absence of PTEN proteolysis could be due to factors linked to LC/E-16x activity as the protease enters the neuronal cytosol via the entry pathway of BoNTs, i.e., entering from the cell outside rather than being expressed directly in the neuronal cytoplasm as previously done (Blum et al. 2021): (i) the LC/E-16x cargo impedes, unlike LC/E-wt, the BoNT translocation machinery; (ii) mutations present in LC/E-16x change the susceptibility to low pH altering the conformational change required for translocation, making it unproductive or highly inefficient; (iii) the amount of catalytically active LC/E-16x reaching the neuronal cytosol is too low or the catalytic activity of LC/E-16x is simply insufficient to generate detectable amounts of cleaved PTEN; (iv) the relative amount of PTEN expressed in the neuronal compartment accessible to the LC/E-16x is insufficient to allow for detection by Western blotting. The interference of LC/E-16x with the BoNT translocation process could be excluded by generating a fusion protein consisting of LC/E-16x fused to an active BoNT/D vehicle and by testing the VAMP-2 cleavage activity. Evidence against LC/E-16x remaining unfolded in the cytosol after translocation are identical yields of soluble LC/E-16x and all intermediate LC/E mutants compared to LC/E-wild-type, i.e. LC/E-16x is stable as isolated protein. Therefore, we speculate that the main reason for undetectable PTEN hydrolysis depends on an insufficient proteolytic activity of the LC/E-16x copies delivered inside the neuronal cytosol. Despite any of the mentioned reasons may contribute to such an insufficient PTEN cleavage, this possibility is in agreement with the observation that, in the test tube, cleavage of PTEN by LCE-16x is achieved in the 30–300 nM range while cleavage of SNAP-25 by LC/E-wt is already substantial at 0.3 nM, i.e., more than two magnitudes of order lower.

Therefore, further improvement of the PTEN specific proteolytic activity seems to be required, recalling the about factor 1000 higher EC_50_ for PTEN (Table 1) versus SNAP-25 (Table 2) cleavage by LC/E-16x and LC/E-wt, respectively. It is impossible to judge to what level the PTEN cleavage activity has to be improved. Future research could lead to an increase in activity by rational replacement of further LC amino acids identified after the clarification of the co-crystal structure of PTEN bound to LC/E-16x whose proteolytic activity was deactivated by mutation of one or two specific amino acids similar to previous approaches (Breidenbach and Brunger 2004). During those studies, one has to ensure that those mutations which abrogate SNAP-25 cleavage, such as E159L and S163R (Table 2), remain untouched. If such an approach succeeds, further aspects have yet to be studied like possible effects on the duration of activity, the identification of the most suitable inactive BoNT as delivery vehicle etc.

In conclusion, the present LC/E-16x does probably not exhibit sufficient proteolytic activity to PTEN when applied as full-length BoNT/E-16x under physiologically relevant concentrations. In addition, a replacement transport vehicle may be required for its delivery in the neuronal cytosol as several of the assembled mutations are likely to interfere with the BoNT/E inherent translocation mechanism. Thus, further optimization of the PTEN-specific LC/E mutant will be key before the next steps in the development of BoNT/E into a novel pharmaceutical can be taken.

## Abbreviations

BoNT: Botulinum neurotoxin
hSNAP-23: Human 23,000 dalton isoform of SNAP-25
LC: Light chain
SNAP-25: Synaptosomal-associated protein of 25 kDa
SNARE: Soluble N-ethylmaleimide-sensitive factor attachment protein receptor
PTEN: Phosphatase and tensin homolog
